# Comparative Expression Analyses of Pro- versus Anti-Inflammatory Mediators within Synovium of Patients with Joint Trauma, Osteoarthritis, and Rheumatoid Arthritis

**DOI:** 10.1155/2017/9243736

**Published:** 2017-02-20

**Authors:** Mohammed A. Al-Madol, Mohammed Shaqura, Thilo John, Rudolf Likar, Reham Said Ebied, Michael Schäfer, Shaaban A. Mousa

**Affiliations:** ^1^Department of Anaesthesiology and Intensive Care Medicine, Charité University Berlin, Campus Virchow Klinikum and Campus Charité Mitte, Berlin, Germany; ^2^Department for Orthopedic and Trauma Surgery, DRK Kliniken Berlin Westend, Berlin, Germany; ^3^Departments of Anaesthesiology and Intensive Care, Hospital Klagenfurt, Klagenfurt, Austria; ^4^Department of Anesthesiology, Theodor Bilharz Research Institute, Imbaba 30, Giza, Egypt

## Abstract

Synovial injury and healing are complex processes including catabolic effects by proinflammatory cytokines and anabolic processes by anti-inflammatory mediators. Here we examined the expression of pro- versus anti-inflammatory mediators in synovium of patients with diagnostic arthroscopy (control), joint trauma (JT), osteoarthritis (OA), and rheumatoid arthritis (RA). Synovial samples from these patients were subjected to RT-PCR and double immunofluorescence confocal microscopy of pro- and anti-inflammatory mediators as well as immune cell markers. Interestingly, pro- and anti-inflammatory mediators were expressed predominantly in granulocytes in patients with JT and in macrophages, lymphocytes, and plasma cells in patients with OA and RA. Interestingly, parallel to the severity of inflammation, proinflammatory mediators IL-1*β*, TNF-*α*, and 5-LOX specific mRNA as well as immunoreactive (IR) cells were significantly more abundant in patients with RA and JT than in those with OA. However, anti-inflammatory mediators 15-LOX, FPR2, and IL-10 specific mRNA as well as IR cells were significantly more abundant in patients with OA than in those with JT and RA. These findings show that upregulation of proinflammatory mediators contributes to the predominantly catabolic inflammatory process in JT and RA synovium, whereas upregulation of anabolic anti-inflammatory mediators counteracts inflammation resulting in the inferior inflammatory process in OA synovium.

## 1. Introduction

In inflammatory joint diseases such as joint trauma (JT), osteoarthritis (OA), and rheumatoid arthritis (RA), the inflammatory process within synovial tissue leads to tissue destruction and fibrosis with varying degrees of severity [1–3]. The degree of severity depends on processes that are taking place inside the synovium including catabolic effects by proinflammatory cytokines concomitant with anabolic processes by anti-inflammatory mediators [4]. The most important group controlling the state of disease seems to be inflammatory cytokines including interleukin-1 *β* (IL-1*β*) and tumor necrosis factor-alpha (TNF-*α*). Tumor necrosis factor-alpha (TNF-*α*), together with IL-1*β*, is considered a key inflammatory cytokine involved in the pathophysiological processes occurring in the course of chronic disease of human synovium. Also, the lipoxygenase isoform of 5-lipoxygenase (5-LOX) is reported to be overexpressed in synovial tissue of patients suffering from rheumatoid arthritis and osteoarthritis. The 5-LOX is involved in the progress of inflammation [5].

The other group opposing these proinflammatory effects in synovial tissue is formed by anti-inflammatory mediators such as 15-LOX, formyl peptide receptor (FPR2), and IL-10 [6, 7]. Previous studies demonstrated that 15-LOX metabolites have potent anti-inflammatory actions on rheumatoid inflammation [8]. FPR2, also known as the lipoxin A4 receptor (ALX), belongs to the Gi-protein coupled receptor (GiPCR) family and is activated by lipoxin A4, Annexin A1, and Annexin A1-derived peptides or by the acute phase reactant serum amyloid A resulting in potent anti-inflammatory effects [9, 10]. Similarly, IL-10 inhibits chemokine and cytokine production from macrophages and dendritic cells that leads to a limitation of the acute and chronic inflammation [11–13]. The exact cell types and disease-specific differences in the occurrence of pro- versus anti-inflammatory mediators within human synovium of patients with JT, OA, and RA have not been examined so far.

Therefore, we investigated the expression of proinflammatory (IL-1*β*, TNF-*α*, and 5-LOX) and anti-inflammatory (15-LOX, FPR2, and IL-10) mediators in synovium of patients with JT, OA, and RA. We assessed the differences in the number and types of cells expressing proinflammatory and anti-inflammatory mediators by the use of different markers for synovial fibroblasts (P4HB), macrophages/monocytes (CD68), granulocytes (CD15), T lymphocytes (CD3), and plasma cells Ab-1. This study may provide conclusive evidence of a disease-specific profile of proinflammatory versus anti-inflammatory mediators as well as cell types involved that may contribute to a better understanding of these opposing mechanisms and to potential targets for the modulation of these processes.

## 2. Materials and Methods

### 2.1. Patients and Synovial Sample Collection

Patients were recruited in three different hospitals: DRK Clinic Westend Berlin, Landeskrankenhaus Klagenfurt, and the University Hospital Regensburg. IRB approval was obtained from all three locations and patients gave their written consent to the participation in this study after they were informed of its purpose (EA). Patients were included when they were scheduled for surgery and had a diagnosis of osteoarthritis, rheumatoid arthritis (according to the criteria of the American College of Rheumatology and to the clinical and radiological criteria of OA) [14, 15], or joint trauma. Patients undergoing a diagnostic arthroscopy were chosen as a control. During surgery, synovial tissues were taken from patients of all four groups and stored at −80°C.

### 2.2. RT-PCR

Frozen synovial tissue samples were homogenized, and then the total RNA was isolated using the Kit Qiazol Lysis Reagent (Qiagen, Hilden, Germany) according to the manufacturer's protocol. For cDNA synthesis, 500 ng total RNA was isolated by NanoDrop (Peqlab). Then, 500 ng total RNA was converted to cDNA at 42°C for 1 h using Omniscript RT Kit (Qiagen, Hilden, Germany) by incubation with 0.5 mM dNTP, 1 *μ*M Random Primer, 10 units RNase Inhibitor, and 4 units Omnisript reverse Transcriptase. The obtained DNA was stored at −20°C. The following specific primers for IL-1*β*, TNF-*α*, 5-LOX, 15-LOX, FPR2, IL-10, and 18s were used (see primers' information in Supplemental Table 1 (see Supplementary Material available online at https://doi.org/10.1155/2017/9243736). Finally, TaqMan® qRT-PCR was performed using a SYBR® Green kit according to the manufacturer's instructions (Applied Biosystems). Amplification was executed with 40 cycles, each consisting of 15 s at 95°C and of 30 s at 60°C. To detect fluorescence specific products for each primer pair, the reaction was carried out at a temperature just below the specific melting temperature (Tm). For statistical analysis, experiments were performed in triplicate in order to determine TNF-*α*, IL1*β*, 5-LOX, 15-LOX, and FPR2 mRNA by the delta-delta CT method as described previously [16].

### 2.3. Tissue Preparation and Histological Evaluation

Intact synovial tissue was fixed in 4% (w/v) paraformaldehyde in 0.16 M phosphate buffer solution (PBS) (pH 7.4) for 4 hours and then cryoprotected overnight at 4°C in PBS containing 10% sucrose. The tissue was then embedded in tissue-Tek compound (OCT, Miles Inc. Elkhart, Indiana) and frozen. For histology, 8 *μ*m sections were cut by using a Cryostat (Thermo Fisher, Dreieich, Germany) and were mounted onto gelatin-coated slides. Histological evaluation of intact synovial tissue was performed as previously described [17], identifying the components of lining cell layers, sublining cells. Cell density was determined in 3 fields in each section of at least 4 different tissue samples from each group (hematoxylin-eosin stained) by counting all stained cell nuclei within 3 randomly selected high-power fields of view per section (400x magnification). The lining-layer thickness was determined from at least three sections and analyzed by averaging the number of cells of three fields per section (400x magnification). The number of B cells, macrophages, fibroblast-like cells, granulocytes, and lymphocytes were evaluated in cryosections stained with antibodies against human plasma cells Ab-1, macrophages (CD68), fibroblasts (P4HB), granulocytes (CD15), and lymphocytes (CD3), respectively. The number of stained structures was averaged from 12 randomly selected high-power fields (400x magnification). The number of high-power fields was derived from a histological study by Bresnihan et al., (1998) [18].

### 2.4. Single and Double Immunofluorescence Staining Procedures

Immunofluorescence staining was processed as described previously [19]. The mounted slide tissue sections were incubated overnight at 4°C with the following primary antibodies (see antibodies' information in supplemental Table 1): single immunofluorescence staining was applied in the following way: (1) anti-IL-1*β*, (2) anti-TNF-*α*, (3) anti-5-LOX, (4) anti-15-LOX, (5) anti-FPR2, and (6) anti-IL-10, respectively, in each group (Control, JT, OA, and RA). Double immunofluorescence staining was performed in RA samples in the following way: (1) anti-TNF-*α*/anti-IL-1*β*, (2) anti-5-LOX/anti-FPR2, (3) anti-15-LOX/anti-5-LOX, and (4) anti-IL-10/anti-FPR2; in OA samples: (1) anti-5-LOX/anti-FPR2, (2) anti-TNF-*α*/anti-IL1*β*, (3) anti-15-LOX/anti-5-LOX, and (4) anti-IL-10/anti-FPR2; in JT samples: (1) anti-TNF-*α*/anti-IL1*β*, (2) anti-5-LOX/anti-FPR2, (3) anti-15-LOX/anti-5-LOX, (4) anti-IL-10/anti-TNF-*α*, and (5) anti-IL-10/anti-FPR2. Antisera specific for certain immune cell types (anti-CD15, anti-CD68, anti-P4HB, anti-CD3, and anti-Ab-1) were applied together with anti-TNF-*α*, anti-IL1*β*, anti-5-LOX, anti-15-LOX, anti-FPR2, or IL-10 within each group. Then, the slides were washed in PBS and for 2 hours incubated with Alexa Fluor 488 donkey anti-rabbit antibody (Vector Laboratories) or with Alexa Fluor 594 donkey anti-goat or with Alexa Fluor 594 donkey anti-mouse (Invitrogen, Germany) (in single staining). For double staining, slides were incubated with Alexa Fluor 488 donkey anti-rabbit antibody combined with Alexa Fluor 594 donkey anti-goat, Alexa Fluor 594 donkey anti-mouse combined with Alexa Fluor 594 donkey anti-goat, or Alexa Fluor 594 donkey anti-mouse combined with Alexa Fluor 488 donkey anti-rabbit. Finally, the tissues were washed in PBS, and nuclei were stained with DAPI (4′,6-diamidino-2-phenylindole) and then mounted in Vectashield (Vector Laboratories) and imaged on a confocal laser scanning microscope, LSM510, as described previously [19]. Then, the photographs were taken by a confocal microscope (633 nm; Carl Zeiss, Göttingen, Germany) as described in our previous study [20]. To demonstrate specificity of staining, the following controls were included: omission of the primary antisera or the secondary antibodies, as described in our previous studies [19].

### 2.5. Quantification of Immunostaining

The method for quantification of the number of immunoreactive cells has been described previously [19]. Immunoreactive cells were counted using only intact tissue exhibiting optimal morphology to avoid misleading results. The numbers of proinflammatory (IL-1*β*, TNF-*α*, and 5-LOX,) and anti-inflammatory (15-LOX, FPR2, and IL-10) immunoreactive cells were counted by a blinded experimenter in three sections per patient. The value of stained proinflammatory and anti-inflammatory mediators was determined by the formula: stained cells/stained cells of control × 100. Data were obtained from three sections per patient from each group: Control (*n* = 5), JT (*n* = 9), OA (*n* = 11), and RA (*n* = 10).

### 2.6. Statistical Analysis

Data are represented as means ± SEM. Sample comparisons were made using one-way analysis of variance followed by Tukey test in the case of normally distributed data and Kruskal–Wallis analysis of variance on ranks followed by Dunn's test in the case of data not distributed normally. Differences were considered significant if *P* < 0.05. All tests were performed using Sigma Plot 13.0 statistical software.

## 3. Results

### 3.1. Patient Recruitment, Demographics, and Synovial Signs of Inflammation

For this study, a total of 42 patients were screened. Seven patients were excluded during the workup, because tissue samples could not be identified histologically as synovial tissue. The remaining patients were distributed according to their clinical diagnosis among the following groups: Control (*n* = 5), JT (*n* = 9), OA (*n* = 11), and RA (*n* = 10).

Patients' demographics such as patient's age, gender, disease duration, and medications are shown in Table 1. Light microscopic evaluation of patients' synovial tissues for lining-layer thickness, overall cellularity, and vascularity was significantly different for patients with RA but not for patients of the other groups compared to control (*P* < 0.05) (Table 1). Overall synovial cellularity was further characterized in greater detail by immunofluorescent microscopy showing different types of immune cells and fibroblasts. Synovial tissue of patients suffering from OA and RA showed significantly more regional immune cells and fibroblasts than control patients and patients with JT (*P* < 0.05). While in patients with JT synovial granulocytes and macrophages were most prominent, in patients with OA fibroblasts and macrophages were the most prominent, and in patients with RA plasma cells, fibroblasts, and macrophages were the most prominent (*P* < 0.05) (Supplemental Figure 1).

### 3.2. Expression of Synovial Proinflammatory Mediators IL-1*β*, TNF-*α*, and 5-LOX

**Figure 1:**
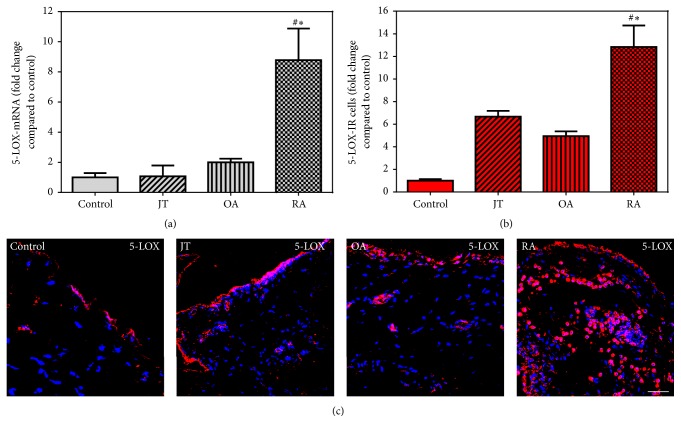
Detection of 5-LOX mRNA (a) and number of 5-LOX-IR cells (b and c) in patients with joint trauma (JT), osteoarthritis (OA), and rheumatoid arthritis (RA). (a) Quantification of 5-LOX mRNA using TaqMan qRT-PCR shows that 5-LOX mRNA was significantly higher in RA compared to JT, OA, and control synovium (*P* < 0.05, one-way ANOVA followed by Tukey's test). (b) Quantitative analysis of immunofluorescence microscopy for 5-LOX-IR cells. ^∗^Relative to control, ^#^relative to other groups (*P* < 0.05, one-way ANOVA followed by Tukey's test). (c) 5-LOX-IR cells are more abundant in RA synovium than in JT, OA, and control synovium. Bar* * = * *20 *μ*m.

Quantitative RT-PCR analysis demonstrated a significant increase in IL-1*β* and TNF-*α* specific mRNA in synovial tissue of JT, OA, and RA patients in contrast to controls. In addition, they were more prominent in JT and RA than in OA patients (Supplemental Figures 2 and 3). Consistently, immunofluorescence confocal microscopy of synovial tissue demonstrated IL-1*β* and TNF-*α* expression in layers of synovial lining and sublining cells. Importantly, the number of IL-1*β*- and TNF-*α*-IR cells was significantly higher in patients with JT, OA, and RA compared to controls (*P* < 0.05) and was more pronounced in JT and RA than OA patients (Supplemental Figures 2 and 3). Quantitative RT-PCR analysis of 5-LOX specific mRNA revealed a significant increase in synovial tissues of RA patients, while immunofluorescence confocal microscopy showed a significant increase of 5-LOX-IR cells in synovial tissues of JT, OA, and RA patients compared to controls which was more prominent in RA patients (*P* < 0.05, Figure 1).

### 3.3. Expression of Synovial Anti-Inflammatory Mediators, FPR2, 15-LOX, and IL-10

**Figure 2:**
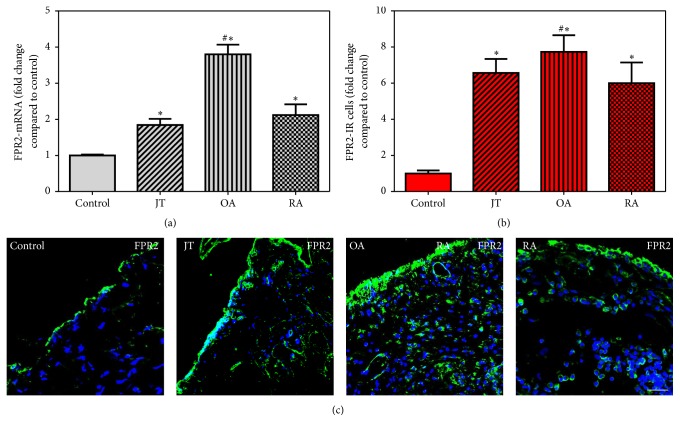
Detection of FPR2 mRNA (a) and number of FPR2-IR cells (b and c) in patients with joint trauma (JT), osteoarthritis (OA), and rheumatoid arthritis (RA). Quantification of FPR2 mRNA (a) and immunofluorescence positive cells (b) shows that FPR2 expression was more prominent in OA compared to RA, JT, OA. ^∗^Relative to control, ^#^relative to other groups (*P* < 0.05, one-way ANOVA followed by Tukey's test). Data are shown as means ± SEM. (c) 5-LOX-IR cells are more abundant in RA synovium than in JT, OA, and control synovium. SEM. Bar* * = * *20 *μ*m.

**Figure 3:**
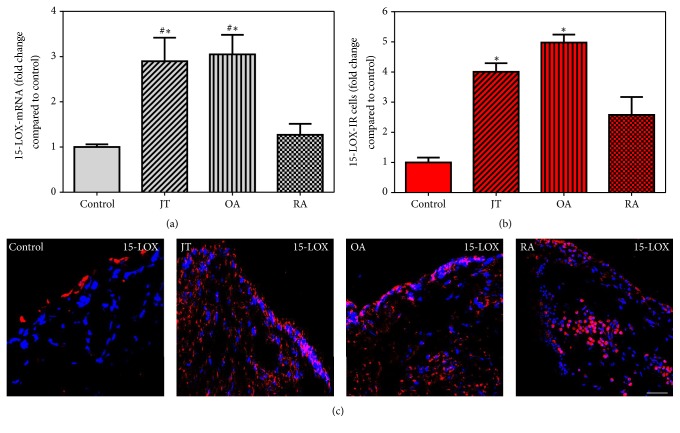
Detection of 15-LOX mRNA (a) and number of 15-LOX-IR cells (b and c) in patients with joint trauma (JT), osteoarthritis (OA), and rheumatoid arthritis (RA). (a) Quantification of 15-LOX mRNA shows that 5-LOX mRNA was significantly higher in OA and JT compared to RA and control synovium (*P* < 0.05, one-way ANOVA followed by Tukey's test). (b) Quantitative analysis of immunofluorescence microscopy for 15-LOX-IR cells. ^∗^Relative to control, ^#^relative to other groups (*P* < 0.05, one-way ANOVA followed by Tukey's test). Data are shown as means ± SEM. (c) 5-LOX-IR cells are more abundant in RA synovium than in JT, OA, and control synovium. Bar* * = * *20 *μ*m.

**Figure 4:**
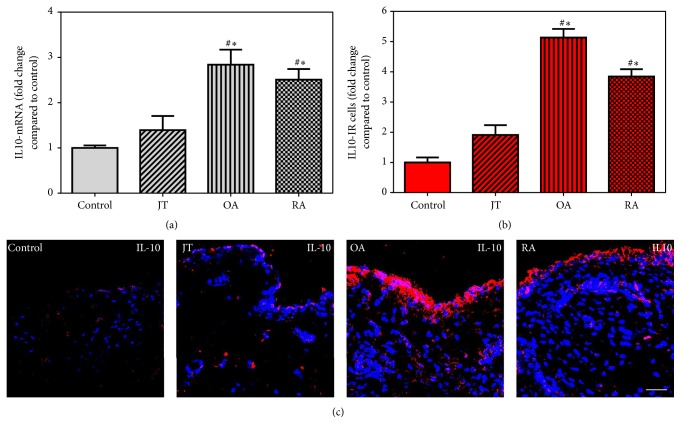
Detection of IL10 mRNA (a) and number of IL10-IR cells (b and c) in patients with joint trauma (JT), osteoarthritis (OA), and rheumatoid arthritis (RA). (a) Quantification of IL10 mRNA shows that IL10 mRNA was significantly higher in RA and OA compared to JT and control synovium (*P* < 0.05, one-way ANOVA followed by Tukey's test). (b) Quantitative analysis of immunofluorescence microscopy for IL10-IR cells. ^∗^Relative to control, ^#^relative to other groups (*P* < 0.05, one-way ANOVA followed by Tukey's test). Data are shown as means ± SEM. (c) 5-LOX-IR cells are more abundant in RA synovium than in JT, OA, and control synovium. Bar* * = * *20 *μ*m.

FPR2 specific mRNA showed a significant increase in synovial tissues of JT, OA, and RA patients compared to controls which was most prominent in OA patients (Figure 2), while 15-LOX mRNA was shown only in JT and OA (Figure 3). However, IL-10 mRNA was prominent only in OA and RA patients (Figure 4). Consistent with these findings, the number of FPR2-IR cells was significantly higher in JT, OA, and RA patients than that of control synovium (Figure 2). However, the number of 15-LOX-IR cells was more prominent in JT and OA than that of RA patients and control synovium (Figure 3), while the number of IL-10-IR cells was higher in RA and OA patients than that of JT and control synovium (Figure 4).

Double immunofluorescence confocal microscopy showed that FPR2 was expressed in CD68-IR macrophages and P4HB-IR fibroblasts in JT, OA, and RA as well as in Ab-1 plasma cells in RA (Supplemental Figure 4). 15-LOX-IR cells were characterized as CD68-IR macrophages and P4HB-IR fibroblasts in OA (Supplemental Figure 5). IL-10-IR cells were mainly CD68-IR macrophages and P4HB-IR fibroblasts in OA and in RA as well as in Ab-1 plasma cells in RA (Supplemental Figure 6).

### 3.4. Inflammatory Mediator Profile in JT, OA, and RA

**Figure 5:**
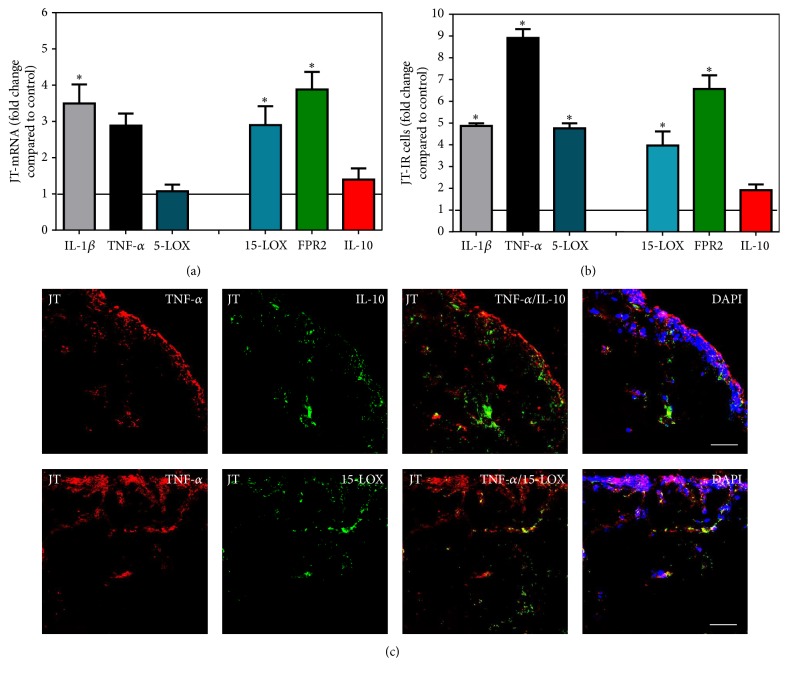
Detection of mRNA (a) and the number of positive cells of inflammatory (IL-1*β*, TNF-*α*, and 5-LOX) versus anti-inflammatory (15-LOX, FPR2, and IL-10) mediators (b and c) in patients with joint trauma (JT). (a) Quantification of mRNA of pro- versus anti-inflammatory mediators shows that there is a balance between expressions of pro- versus anti-inflammatory mediators in JT synovium. (b) Quantitative analysis of immunofluorescence microscopy of pro- versus anti-inflammatory mediators shows that the number of cells expressing anti-inflammatory mediators was comparable with those containing proinflammatory mediators in JT synovium. Data are shown as means ± SEM. ^∗^Relative to control (*P* < 0.05, one-way ANOVA followed by Tukey's test). (c) Confocal microscopy of pro- (*red fluorescence*; (a), (b)) and anti-inflammatory mediators (*green fluorescence*; (c), (d)) double immunofluorescence (e–h) in JT synovium. Note that anti-inflammatory mediator expression was comparable with those containing inflammatory mediators in JT synovium. Bar = 40 *μ*m.

**Figure 6:**
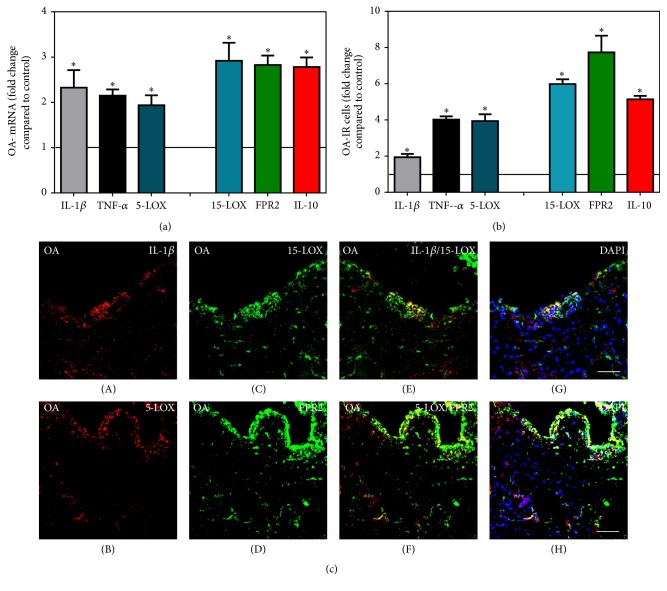
Detection of mRNA (a) and the number of positive cells of pro- (IL-1*β*, TNF-*α*, and 5-LOX) versus anti-inflammatory (15-LOX, FPR2, and IL-10) mediators (b and c) in patients with osteoarthritis (OA). Quantification of mRNA of pro- versus anti-inflammatory mediators shows that anti-inflammatory mediator expression was more prominent than inflammatory mediators in OA synovium. ^∗^Relative to control (*P* < 0.05, one-way ANOVA followed by Tukey's test). (b) Quantitative analysis of immunofluorescent microscopy pro- versus anti-inflammatory mediators shows that the number of cells expressing anti-inflammatory mediators was more prominent than those containing proinflammatory mediators in OA synovium; the asterisks denote significant differences (*P* < 0.05, one-way ANOVA followed by Dunn's test). Data are shown as means ± SEM. (c) Confocal microscopy of pro- (*red fluorescence*; (A) and (B)) and anti-inflammatory mediators (*green fluorescence*; (C) and (D)) double immunofluorescence in OA synovium. Note that anti-inflammatory mediator expression was more prominent than proinflammatory mediators in OA synovium. Bar = 40 *μ*m.

**Figure 7:**
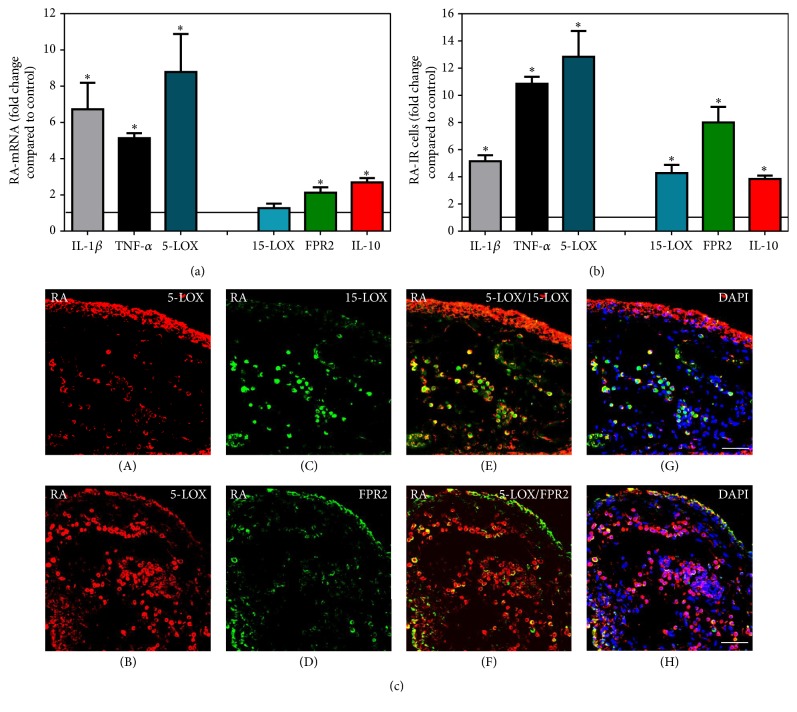
Detection of mRNA (a) and the number of positive cells of pro- (IL-1*β*, TNF-*α*, and 5-LOX) versus anti-inflammatory (15-LOX, FPR2, and IL-10) mediators (b and c) in patients with rheumatoid arthritis (RA). Quantification of mRNA of pro- versus anti-inflammatory mediators shows that proinflammatory mediators expressions were more prominent than anti-inflammatory mediators in RA synovium. ^∗^Relative to control (*P* < 0.05, one-way ANOVA followed by Tukey's test). (b) Quantitative analysis of immunofluorescence microscopy pro- versus anti-inflammatory mediators shows that the number of cells expressing proinflammatory mediator expression was more prominent than those containing anti-inflammatory mediators in OA synovium; the asterisks denote significant differences (*P* < 0.05, one-way ANOVA followed by Dunn's test). Data are shown as means ± SEM. (c) Confocal microscopy of pro- (*red fluorescence*; (A) and (B)) and anti-inflammatory mediators (*green fluorescence*; (C) and (D)) double immunofluorescence in RA synovium. Note that proinflammatory mediator expression was more prominent than anti-inflammatory mediators in RA synovium. Bar = 40 *μ*m.

Direct comparison of the mRNA expression profile of pro- versus anti-inflammatory mediators in synovial tissues revealed that cells expressing the proinflammatory mediators IL-1*β*, TNF-*α*, and 5-LOX were elevated approximately 4- to 8-fold compared to controls in JT (Figure 5), whereas they were upregulated only 2- to 4-fold in OA (Figure 6). At the same time, the anti-inflammatory mediators 15-LOX, FPR2, and IL-10 were found to be elevated approximately 2- to 6-fold in JT (Figure 5), whereas in OA they were elevated approximately 5- to 7-fold (Figure 6). In contrast, in synovial tissue of RA patients, the proinflammatory mediators IL-1*β*, TNF-*α*, and 5-LOX were elevated approximately 5- to 13-fold compared to controls, whereas the anti-inflammatory mediators 15-LOX, FPR2, and IL-10 were upregulated approximately 3- to 7-fold (Figure 7).

## 4. Discussion

We found the highest amount of synovial lining cells, vascularity, and infiltrating immune cells in patients with RA compared to those with JT and OA. Pro- and anti-inflammatory mediators were expressed predominantly in granulocytes and macrophages in patients with JT and in macrophages, fibroblasts, and plasma cells in those with OA and RA. Overall, proinflammatory IL-1*β*, TNF-*α*, and 5-LOX specific mRNA as well as immunoreactive cells were significantly more abundant in patients with RA and JT than in those with OA. In contrast, anti-inflammatory 15-LOX, FPR2, and IL-10 specific mRNA as well as immunoreactive cells were significantly more abundant in patients with OA than in those with JT and RA. These findings provide a morphological evidence of imbalance within the so-called inflammatory factor network between catabolic proinflammatory and anabolic anti-inflammatory mediators among JT, OA, and RA patients.

The lining layer of the synovial membrane contains macrophage-like (type A cells) and fibroblast-like (type B cells) cells [19, 21, 22]. Our double staining demonstrated that most of the lining-layer cells containing proinflammatory cytokines such as IL1*β*, TNF-*α*, and 5-LOX were positive for CD68 (macrophages), CD15 (granulocytes), and P4HB (fibroblast-like synoviocytes, type B) [19]. In the sublining layers, we found different patterns of pro- versus anti-inflammatory mediators containing leukocyte subsets in patients with JT, OA, and RA. Pro- and anti-inflammatory mediators were expressed predominantly in granulocytes in patients with JT and in macrophages, lymphocytes, and plasma cells in those with OA and RA. These results extend previous reports of the expression of 5-LOX in macrophages, fibroblasts, and neutrophils [5] and TNF-*α* in macrophages and monocytes [23] within RA synovium.

Importantly, our results showed that IL-1*β* and TNF-*α* and 5-LOX specific mRNA as well as cells expressing these mediators within synovial tissue were more prominent in JT and RA compared to OA patients. These findings are consistent with high levels of 5-LOX reported to be present in RA synovium and mostly expressed in macrophages, neutrophils, and mast cells of the sublining layer [5]. Also, our results are in agreement with the previous study by Park and Pillinger [24], suggesting that the inflammatory mediators such as TNF-*α*, IL-1*β*, and 5-LOX play a key role in driving the inflammation and synovial cell proliferation in RA associated joint destruction. Taken together, these results suggest that there is an association between the expression of proinflammatory mediators such as TNF-*α*, IL-1*β*, and 5-LOX and the severity of inflammation among patients with JT, OA, and RA [19].

On the other hand, the present results showed that the anti-inflammatory cytokines 15-LOX, FPR2, and IL-10 specific mRNA as well as immunoreactive cells were more abundant in patients with OA than in those with JT and RA. The present results extend the previous reports of 15-LOX expression in humans [5] that did not differentiate the synovial cell types. IL-10 is a potent immunoregulatory cytokine and plays a role in preventing exaggerated inflammatory and immune responses and thus protects the host from immune-mediated damage [25]. Moreover, IL-10 is a good candidate transgene to suppress arthritis using disease-regulated promoters. Also, IL-10 is a broad spectrum anti-inflammatory cytokine and is produced by different immune cells, like Th1 and Th2 cells, B cells, monocytes, and macrophages [11–13]. Inhibition of several proinflammatory cytokines has been reported; effects were seen for interleukin-1 (IL-1) and TNF-*α* [26, 27]. Recently, Vermeij et al. [28] showed that treatment of an acute joint inflammation with local IL-10 overexpression under the control of disease-regulated promoters inhibited arthritis progression. Consistently, Roybal et al. [29] showed that the early gestational gene transfer of IL-10 by systemic administration of lentiviral vector can prevent arthritis in a murine model. Taken together, these findings are consistent with the notion that the upregulated proinflammatory mediators support the inflammatory process in JT and RA synovium in contrast to the upregulated anti-inflammatory mediators probably responsible for a counterbalance and, therefore, lower inflammatory process in OA synovium. A potential limitation of this study, however, is that the observed differences, at least in part, may be also due to apparent differences in age and disease duration of patients influencing the disease-specific pro- and anti-inflammatory balance (see Table 1).

In summary, we found that the upregulation of proinflammatory mediators mediates the predominantly catabolic inflammatory process in JT and RA synovium, whereas the upregulation of anabolic anti-inflammatory mediators may counteract inflammation and be responsible for the inferior inflammatory process in OA synovium. These findings provide a morphological evidence of imbalance within the so-called inflammatory factor network between catabolic proinflammatory and anabolic anti-inflammatory cytokines within each disease and among JT, OA, and RA patients.

## Supplementary Material

The supplementary material gives further information on the specifications of all antisera used in this study (supplemental table 1), on the divers cellularity of synovial tissue (suppl. figure 1), on the synovial mRNA expression and immunoreactive cells of IL-1β (suppl. figure 2) and TNF-α (suppl. figure 3), as well as on the FPR2- (suppl. figure 4), 15-LOX- (suppl. figure 5), and IL-10- (suppl. figure 6) immunoreactivity in distinct cell types of synovial tissue of patients with JT, OA and RA compared to control.

## Figures and Tables

**Table 1:** Clinical and histological characteristics of patients with joint trauma, osteoarthritis, and rheumatoid arthritis.

Patients	Control	JT	OA	RA
	(*n* = 5)	(*n* = 9)	(*n* = 11)	(*n* = 10)
Age (years)	65 (±16,8)	48 (±17)	72 (±6)	64.9 (±17)
Sex (F/M)	1/4	9/6	5/2	6/4
Disease duration:				
≤1 year	5/5	4/9	2/11	—
>1 year	—	5/9	9/11	10/10
Drugs; NSAIDs: (Diclofenac, Ibuprofen, Profenid, Paracetamol, Dipyrone)	5/5	8/9	8/11	8/10
Etoricoxib	NA	1/9	3/11	1/10
Prednisolone (Dexamethasone)	NA	NA	NA	7/10
Lining-layer thickness (cell-layers)	1 (1; 2)	2 (1; 3)	3 (3; 4)	4 (3; 5)
Overall cellularity (cells/mm^2^)	47 (44; 49)	124 (97; 138)	269 (220; 285)	387 (358; 474)
Vascularity (vessels)	2 (1; 3)	3 (3; 5)	6 (5; 7)	6 (3; 8)
