# Supplementary material for: Comparative Expression Analyses of Pro- versus Anti-Inflammatory Mediators within Synovium of Patients with Joint Trauma, Osteoarthritis, and Rheumatoid Arthritis
Source: Mediators Inflamm. 2017 Feb 20;2017:9243736. doi: 10.1155/2017/9243736 (PMC5337844; doi:10.1155/2017/9243736)

Supplement Table 1

Antigen	Manufacturer, Species, Type, Catalogue Number	DILUTION
COX-2	Abcam; Cambridge, UK Polyclonal Rabbit IgG Anti-human # ab6665	1:400
FPRL1	Abcam, Cambridge, UK Polyclonal Rabbit IgG Anti-human # ab101702	1:200
EP1, EP2, EP4, EP4	A gift from Dr. R. Nüsing, Institute of clinical pharmacology, Johann Wolfgang Goethe University, Frankfurt, Germany Polyclonal Rabbit IgG Anti-human	1:300
COX-2	BD Transduction Laboratories; MD, USA Monoclonal Mouse IgM Anti-huma # Nr.610203;	1:300
15-LOX	Abnova, Taipei, TaiwanMonoclonal Mouse IgG2a Anti-human # 12080- 3D8	1:100
IL-1β	Santa Cruz Biotechnology, INC. USA polyclonal Goat IgG against human<br>#M-20; SC-1251	1:200
TNFα	Santa Cruz Biotechnology, INC. USA Polyclonal Goat IgG against human # C-20; SC-1347	1.100
5- LOX	Santa Cruz Biotechnology, INC. USA polyclonal Goat IgG against human # N-19; SC-8885	1:200
CD15	Dako; Glostrup. Denmark Monoclonal Mouse IgM against human<br># Clone C3D-1; Nr. M0733	1:200
CD68	Dako; Glostrup. Denmark Monoclonal Mouse IgG against human # M 0814 clone KP1(macrophages)	1:200
Prolyl-4-Hydroxylase beta	Acris Antibodies, Inc., San Diego, CA, USA Monoclonal Mouse IgG1 against human # Clone 3-2B12	1:400
Plasma cell Ab-1	Lab vision, CA, USA Monoclonal Mouse IgG2a against human # Clone LIV3G11	1:500

Supplement Figure 1

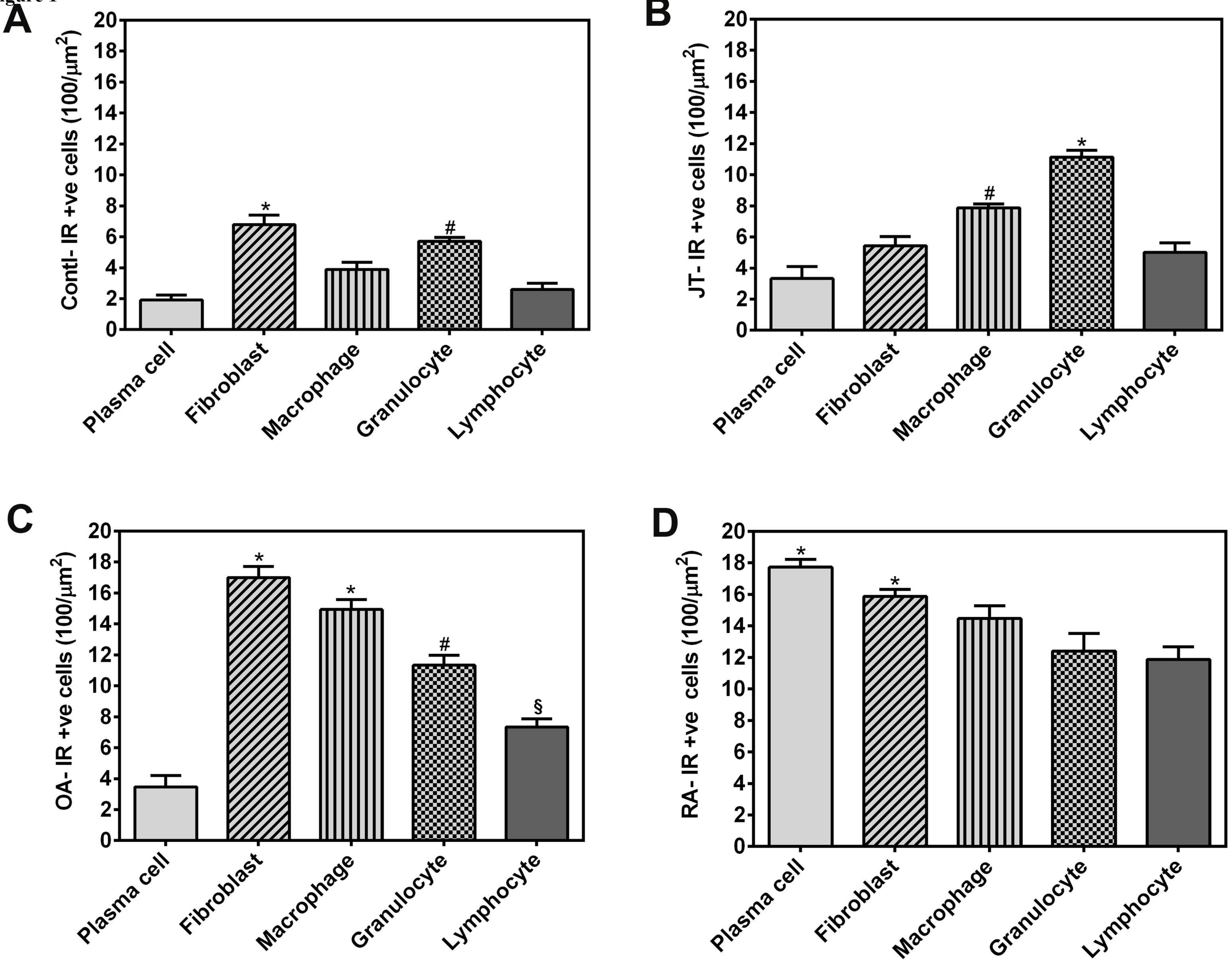

Supplement Figure 2

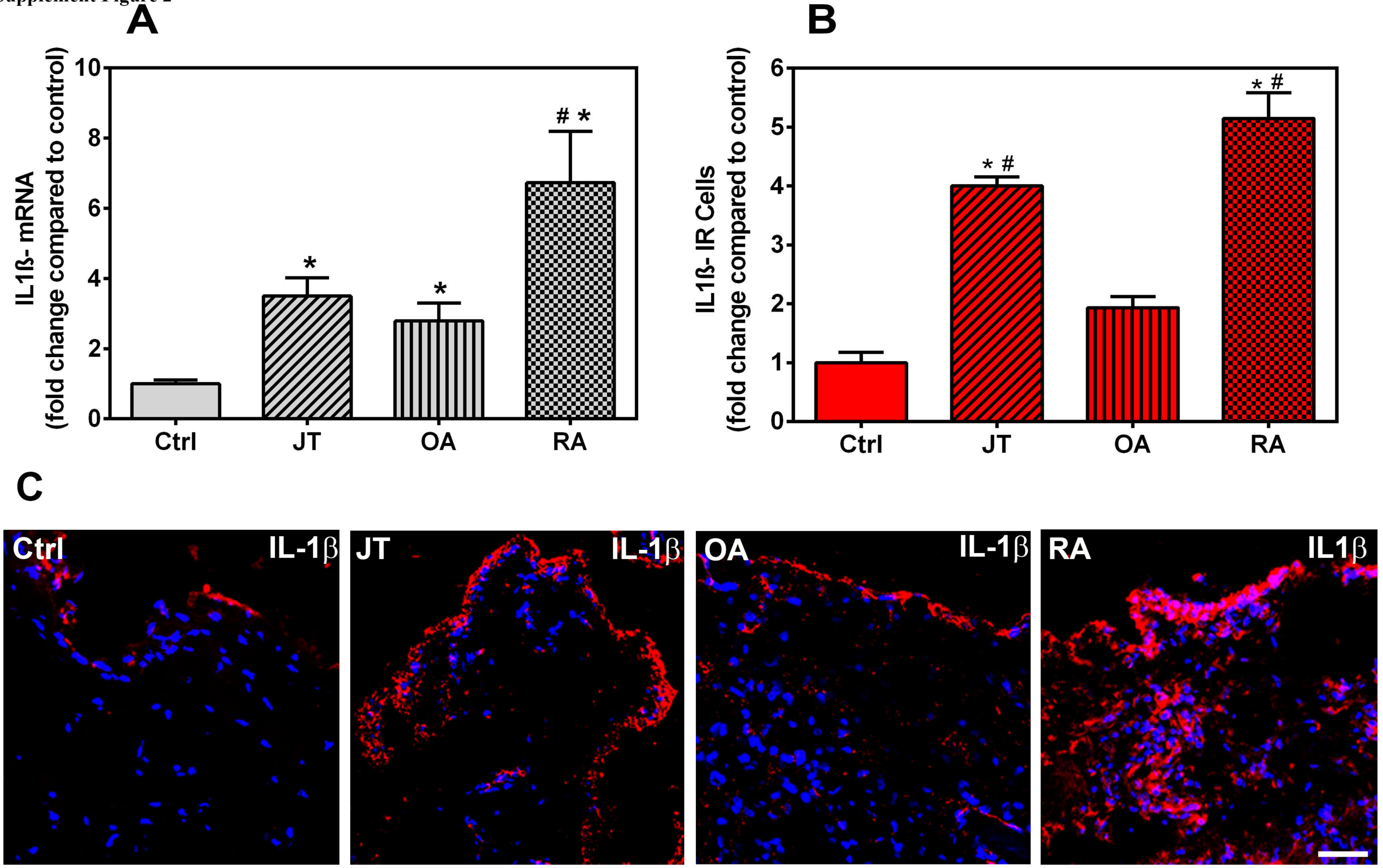

Supplement Figure 3

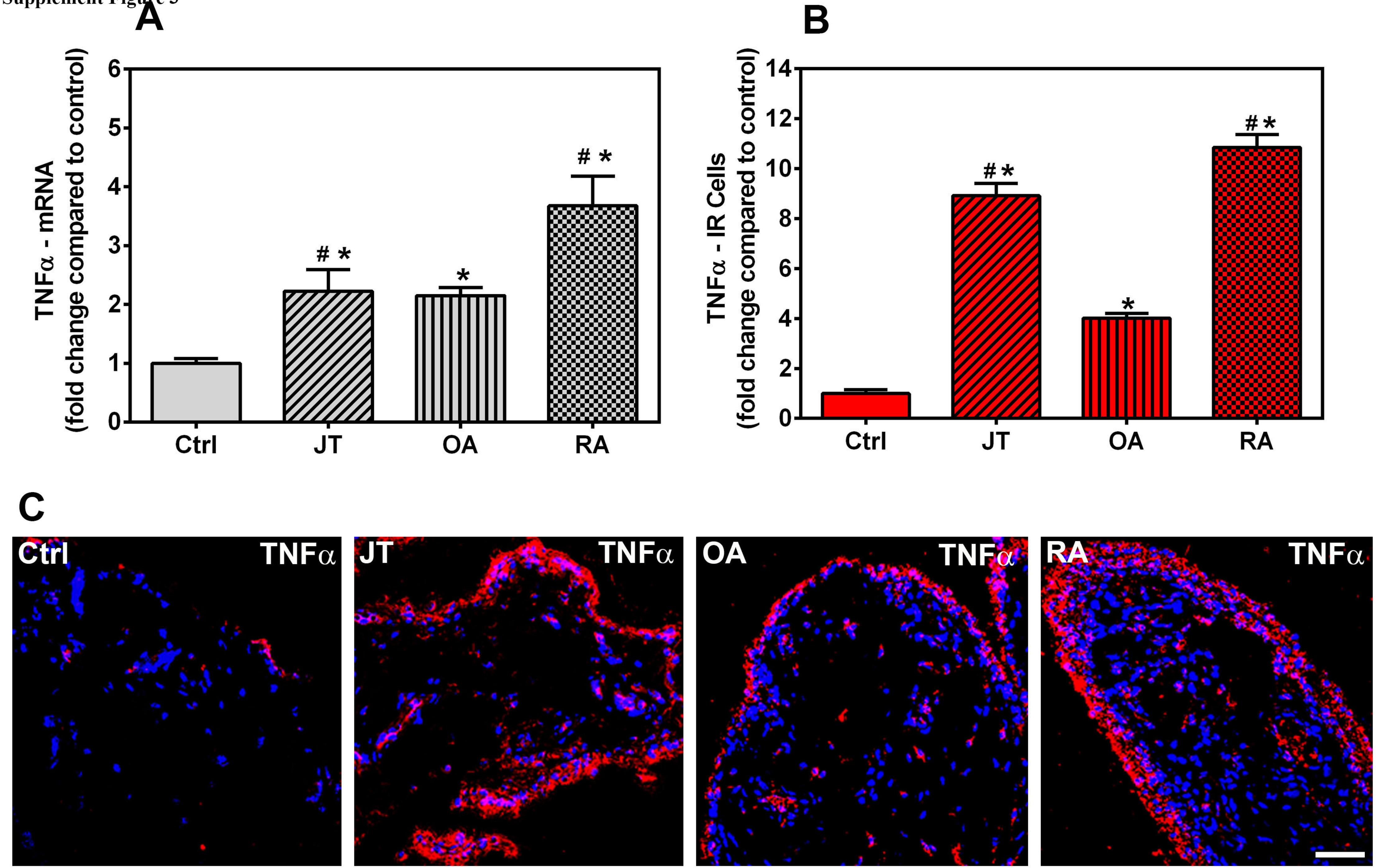

Supplement Figure 4

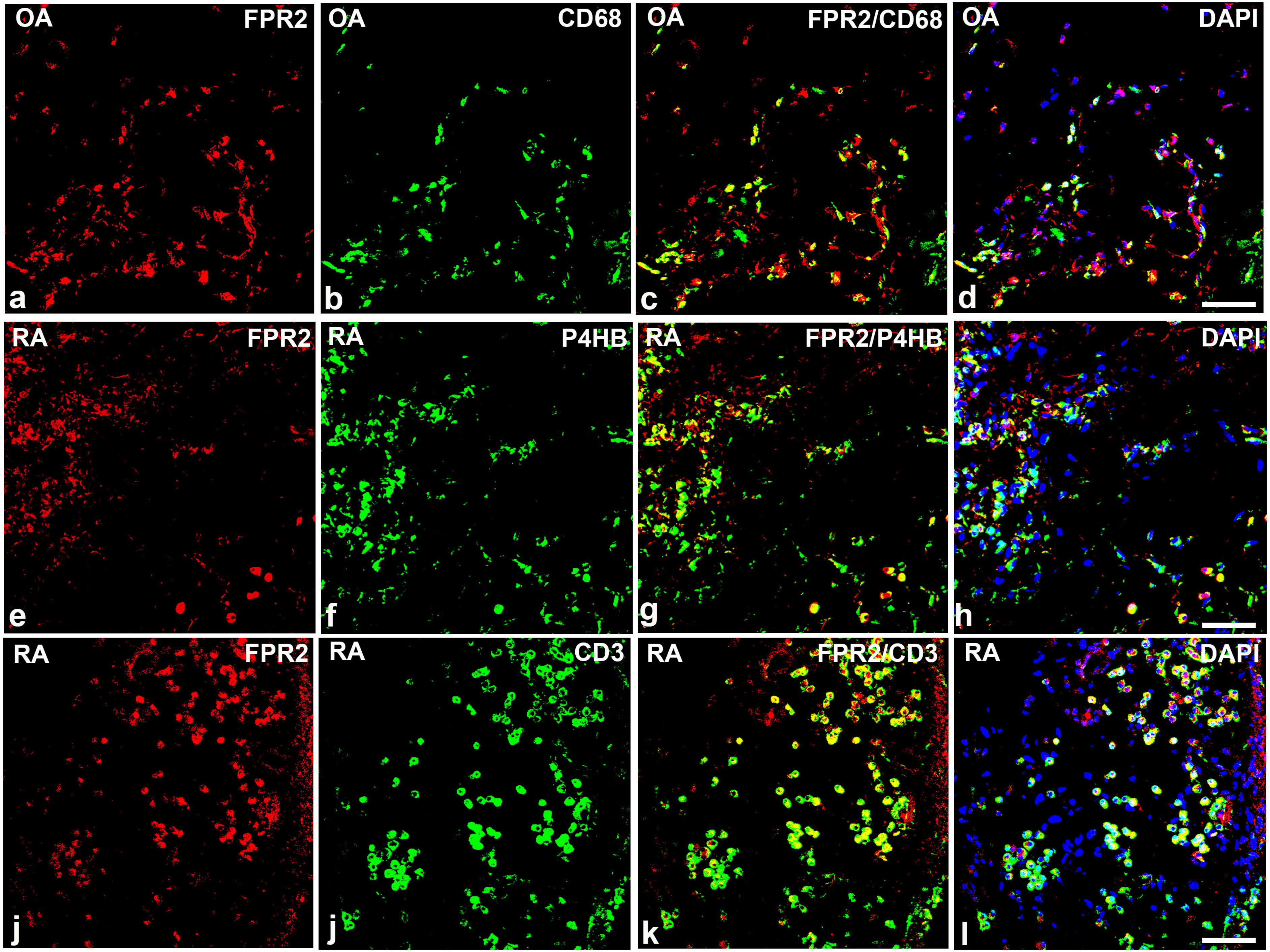

Supplement Figure 5

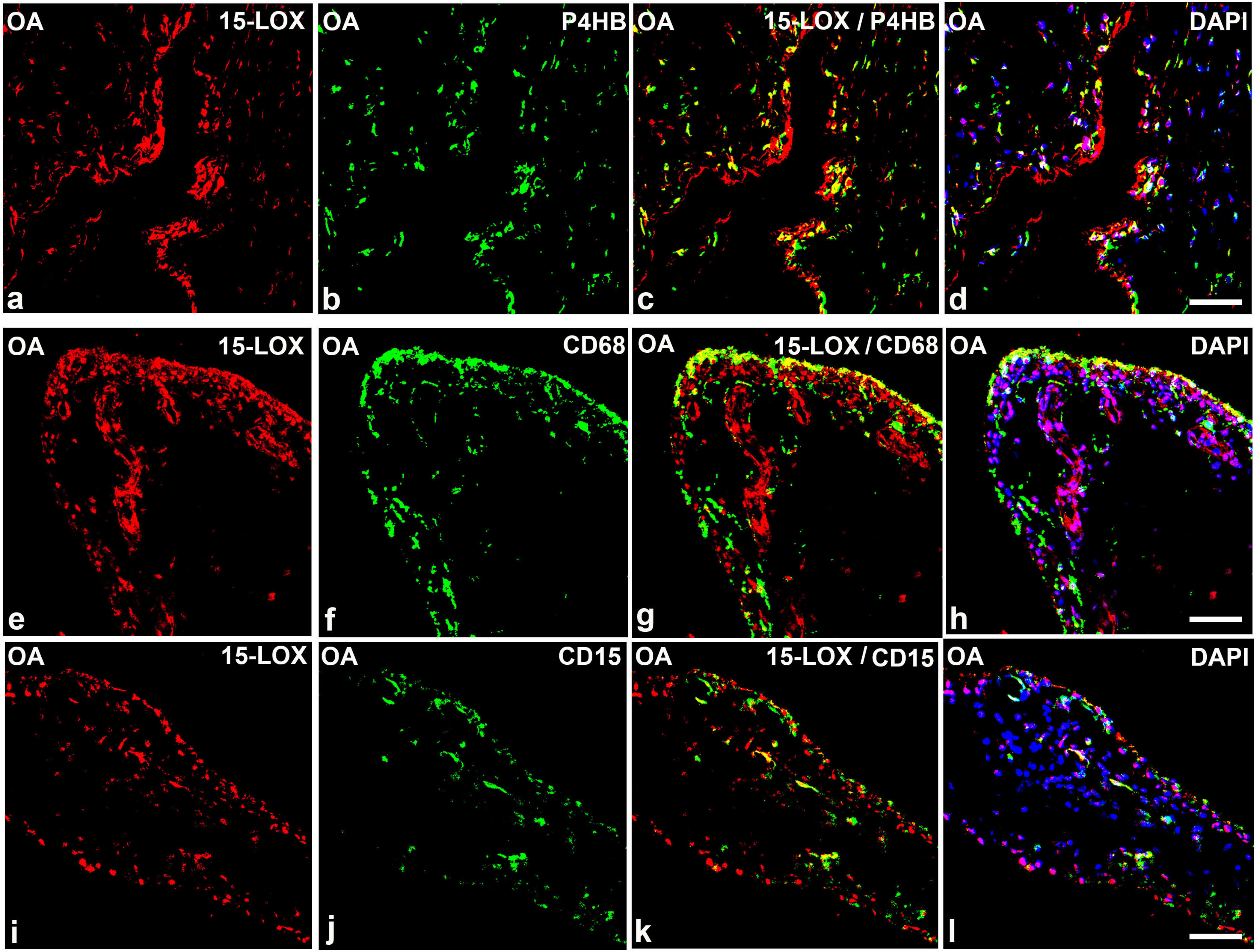

Supplement Figure 6

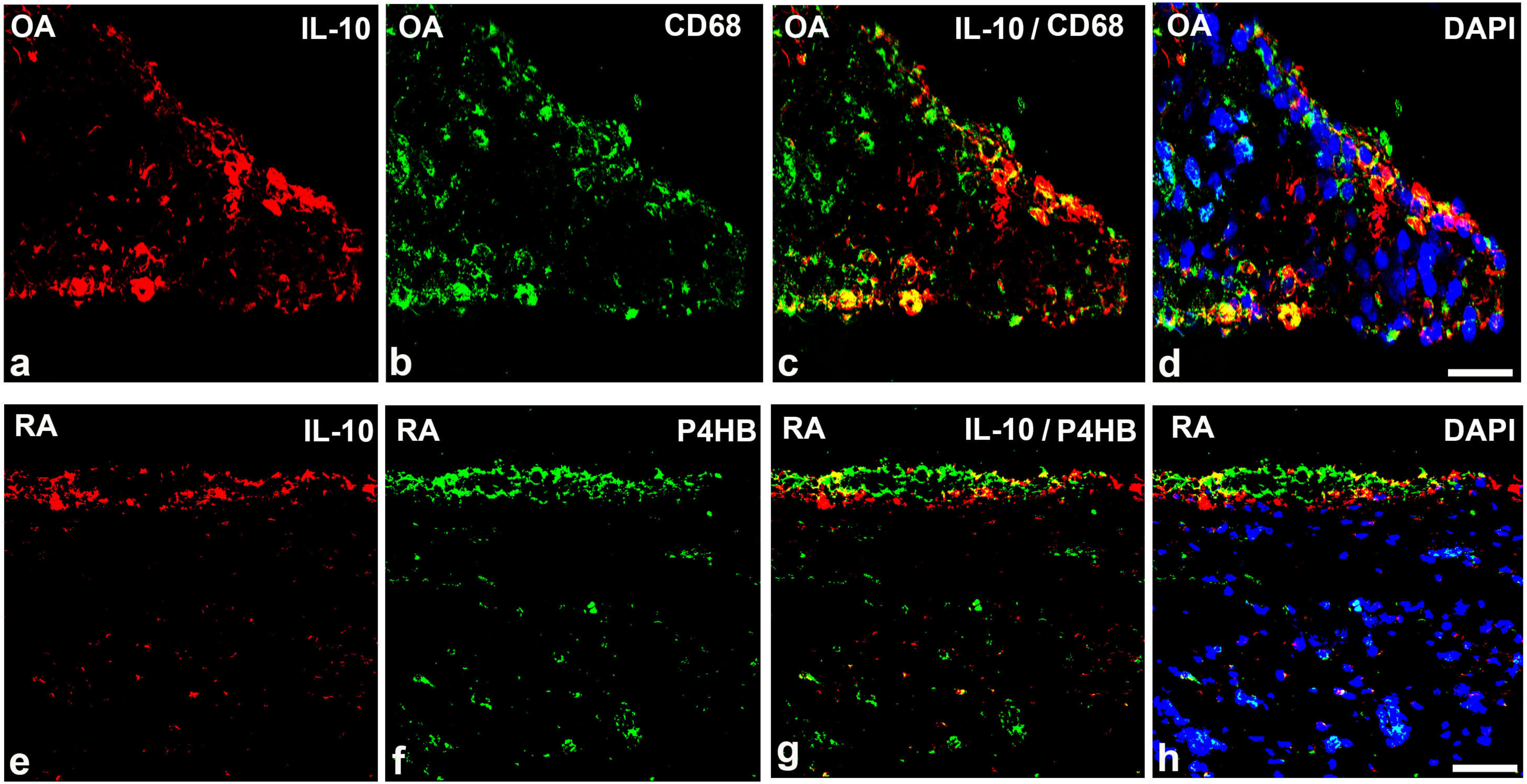

Supplement: Supplementary file 1 — The supplementary material gives further information on the specifications of all antisera used in this study (supplemental table 1), on the divers cellularity of synovial tissue (suppl. figure 1), on the synovial mRNA expression and immunoreactive cells of IL-1β (suppl. figure 2) and TNF-α (suppl. figure 3), as well as on the FPR2- (suppl. figure 4), 15-LOX- (suppl. figure 5), and IL-10- (suppl. figure 6) immunoreactivity in distinct cell types of synovial tissue of patients with JT, OA and RA compared to control. [file 9243736.f1.pdf]
